# Zeolitic imidazolate framework-8 was coated with silica and investigated as a flame retardant to improve the flame retardancy and smoke suppression of epoxy resin†

**DOI:** 10.1039/c7ra12816a

**Published:** 2018-01-11

**Authors:** Wenzong Xu, Guisong Wang, Yucheng Liu, Rui Chen, Wu Li

**Affiliations:** School of Materials Science and Chemical Engineering, Anhui Jianzhu University 292 Ziyun Road Hefei Anhui 230601 People's Republic of China wenzongxu@ahjzu.edu.cn +86-0551-63828157 +86-0551-63828157

## Abstract

A material (ZIF-8@SiO_2_) with a nuclear shell structure was synthesized to improve the flame retardancy and smoke suppression of epoxy resin (EP) through a synergetic catalytic effect. Zeolitic imidazolate framework-8 (ZIF-8) was synthesized and its surface was coated with SiO_2_ by hydrolyzing tetraethyl orthosilicate. Core–shell structured ZIF-8@SiO_2_ was obtained. Then ZIF-8@SiO_2_ was added to EP to explore its effect of flame retardancy and smoke suppression on the EP composite. The results of a cone calorimeter test showed that the peak heat release rate, total heat release, smoke production rate and total smoke production of the material were decreased by 75.9%, 38.9%, 51.1% and 39.8%, respectively, after 2 wt% ZIF-8@SiO_2_ was added to EP. Besides, through the analysis of char residue, the mechanism of ZIF-8@SiO_2_/EP's flame retardancy and smoke suppression was determined to be due to the physical barrier effect of SiO_2_ and the co-effect of SiO_2_ and ZnO decomposition by ZIF-8.

## Abstract 2

Synthesis of the core–shell structure of ZIF-8@SiO_2_ and its effect on the flame retardancy of epoxy resin.

## Graphical abstract

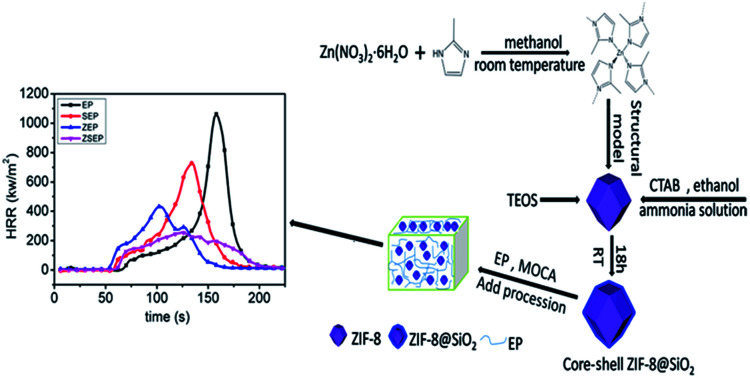

## 1. Introduction

Metal–organic frameworks (MOFs) are porous materials comprising metal ions and organic bridging ligands.^1^ ZIF-8 is a metal–organic framework material with a rhombic dodecahedral structure and is synthesized in organic solvent with zinc ions and 2-methylimidazolate.^2^ Because ZIF-8 has a high specific surface area, high thermal stability and unique pore structure, it is widely applied in adsorption, catalysis, separation, gas storage and drug delivery.^3–8^ Due to the diversity of nanometer ZIF-8 materials, their new functions are being explored. Therefore, the application of ZIF-8 as a flame retardant has also attracted attention. For instance, Shi *et al.*^9^ prepared ZIF-8/PLA composite materials, and their research indicated that the oxygen index of the composite could reach 26.0% when 3 wt% ZIF-8 was added. The application of ZIF-8 as a new flame retardant is worthy of further exploration.

In recent years, the application of silicon-containing compounds in flame retardants has been investigated by many researchers because they not only improve the flame retardant performance, but also form environment-friendly flame retardants.^10^ The combustion of silicon-containing polymer composites may produce SiO_2_ which is easy to migrate and accumulate to the polymer surface, and generate Si–O–Si networks inside the char layer structure and ceramic-like protective material on the substrate surface to prevent the polymer's further burning and protect internal polymer. In addition, some compounds containing Si–C structure are also produced, which enhance the thermal stability of composite materials and increase the strength of the char layer.^11–13^ Meanwhile, SiO_2_ decomposed by silicon-containing compounds is a solid acid with more acidic sites, which can catalyze the degradation of polymers and have a good co-effect of flame retardancy with metal oxides.^14^ According to existing literature,^15^ the use of SiO_2_ coated brucite as a flame retardant has a better effect of flame retardancy compared with the simple physical mixing of SiO_2_ and brucite. Jiang *et al.*^16^ prepared m-SiO_2_@Co–Al LDH to study its effect on EP's flame retardancy performance, and their results showed that the thermal stability and flame retardancy performance of EP composites were significantly improved. Therefore, according to the above, it is assumed that polymer may have a better flame retardancy and smoke suppression effect by adopting the core–shell structure of ZIF-8@SiO_2_.

EP is widely used in coating, adhesives, laminated materials, electronics, *etc.*^17–20^ because cured epoxy resin has good physical and chemical properties. However, the flammability of epoxy resin has limited the further development of its application. The flame retardant of a single component does not achieve the ideal effect of flame retardancy. In addition, as far as we know, there has been no report about the application of ZIF-8@SiO_2_ for the flame retardancy of EP.

This paper presents our study on ZIF-8@SiO_2_. ZIF-8 nanoparticles were first synthesized at room temperature. SiO_2_ was then coated on the surface of ZIF-8 by the sol–gel method. The core–shell structure of ZIF-8@SiO_2_ was duly prepared. The structure and surface microtopography were characterized and analysed. Finally, ZIF-8@SiO_2_ was added to EP to study its effect of flame retardancy and smoke suppression on EP and the mechanism of its action. Scheme S1† presents the process of sample preparation.

## 2. Experimental

### 2.1 Materials

2-Methylimidazolate, Zn(NO_3_)_2_·6H_2_O, absolute methanol, absolute ethanol, tetraethyl orthosilicate (TEOS), ammonium hydroxide and cetyltrimethyl ammonium bromide (TEOS) were purchased from Sinopharm Chemical Reagent Co. Ltd. E-44 epoxy resin was obtained from Changzhou Lebang Composite Materials Corporation. 3,3′-Dichloro-4,4′-diaminodiphenylmethane (MOCA) was obtained from Guangzhou Fufei Chemical Corporation. Deionized water was produced at our laboratory.

### 2.2 Synthesis of SiO_2_ nanoparticles

SiO_2_ nanoparticles were synthesized as in ref. 21. In brief, firstly, 4.5 ml TEOS and 4.5 ml ethanol were mixed, and then 9 ml NH_3_·H_2_O and 16.3 ml ethanol were added to the above solution and stirred for 1 h at room temperature. After that, the suspension was obtained. Finally, the suspension was centrifuged and washed with deionized water and ethanol (1 : 1) mixture for three times and vacuum freeze dried. SiO_2_ nanoparticles were obtained as a result.

### 2.3 Synthesis of ZIF-8 nanocrystals

ZIF-8 nanocrystals were synthesized by means of co-precipitation.^22^ In short, firstly, 2.933 g Zn(NO_3_)_2_·6H_2_O was dissolved in 200 ml methanol and recorded as solution A. 6.489 g 2-methylimidazole was dissolved in 200 ml methanol, and recorded as solution B. Then, the suspension was obtained by mixing solution A and solution B, with magnetic stirring for 1 h at room temperature. Finally, the suspension was centrifuged and washed with methanol for three times and dried under vacuum conditions at 40 °C. ZIF-8 nanocrystals were thus obtained.

### 2.4 Synthesis of core–shell structure of ZIF-8@SiO_2_

Firstly, 1.0 g CTAB was dissolved in 80 ml ethanol. Then 0.8 g ZIF-8 was added to it, whose PH was adjusted to 11 with NH_3_·H_2_O, and the suspension was treated by ultrasonic dispersing technology for 5–10 min. After that, the mixed solution of 4 ml TEOS and 16 ml ethanol was added into the suspension drop by drop and reacted for 18 h at room temperature. Finally, the product was centrifuged and washed with a mixture of ethanol and deionized water (1 : 1) several times and vacuum freeze dried. In the end, core–shell structure of ZIF-8@SiO_2_ was obtained.

### 2.5 Preparation of EP composite

First, ZIF-8@SiO_2_ was evenly distributed in a proper amount of acetone solution, and it was added to epoxy resin under ultrasonication. It was treated with ultrasonication and strong stirring simultaneously until the formation of a homogeneous mixture under 60 °C. Then the molten MOCA was added to the mixture with full stirring to spread evenly, and poured into polytetrafluoroethylene molds to cure at 120 °C for 2 h and at 150 °C for 1 h, respectively. The EP composite was obtained after cooling. SiO_2_ and ZIF-8 were also mixed with epoxy resin under the same conditions (the specific formula for the experiment is shown in Table 1).

**Table 1:** Formulas of neat EP and EP composites

Sample	EP (wt%)	SiO_2_ (wt%)	ZIF-8 (wt%)	ZIF-8@SiO_2_ (wt%)
EP	100	0	0	0
SEP	98	2	0	0
ZEP	98	0	2	0
ZSEP	98	0	0	2

### 2.6 Characterization

X-ray diffraction (XRD) patterns were obtained with a D8X diffractometer (Bruker/AXS Company, Germany) at a scanning speed of 2° min^−1^ and a measurement range of 2*θ* from 6° to 60°. Fourier transform infrared (FTIR) spectra were obtained with a Nicolet 6700 FTIR spectrophotometer (USA) at a test range of 400–4000 cm^−1^ by the KBr pellet technique. Scanning electron microscopy (SEM) and energy dispersive X-ray spectroscopy (EDS) were carried out using a XL-30 scanning electron microscope (Dutch Philips Company). Transmission electron microscopy (TEM) tests were performed to use a JEM-2100 transmission electron microscope (Japan) through direct observation to observe surface the microtopography and size of samples. Before measurement, all the samples were dispersed in ethanol with ultrasonication for a while. Thermogravimetric analysis (TGA) was performed with a German Netzsch 209 F3 thermal analyzer. The samples underwent heating from room temperature to 750 °C under air atmosphere at a heating rate of 20 °C min^−1^. Differential Scanning Calorimetry (DSC) was taken with a TA Q20 (USA). The samples were treated under nitrogen atmosphere at a heating rate of 10 °C min^−1^. Laser Raman Spectroscopy (LRS) was conducted with a LabRam HR spectrometer (JY, France). X-ray photoelectron spectroscopy (XPS) was determined by an AXIS Ultra XPS spectrometer (Kratos, UK) using Al Kα excitation radiation at the power of 225 W (working voltage: 15 kV; transmitting current: 15 mA). The UL-94 burning test was taken with a UL-94 SCZ-3 (China), the samples size was 125 × 13 × 3 mm^3^. The limit oxygen index (LOI) was obtained with an HC-2 oxygen index meter (Jiangning Analytical Instrument Company, China). The samples size of the test was 100 × 10 × 3 mm^3^. The JCZ-2 type cone calorimeter was similar to the one for the LOI in accordance with ISO 5660. The samples size was 100 × 100 × 3 mm^3^, and the heat flux used was 50 kW m^−2^.

## 3. Results and discussion

### 3.1 Characterization of SiO_2_, ZIF-8 and ZIF-8@SiO_2_

#### 3.1.1 XRD characterization

Fig. S1† shows the XRD patterns of SiO_2_, ZIF-8 and ZIF-8@SiO_2_. The diffraction peaks in Fig. S1† are at 6° to 60°. From the XRD patterns of SiO_2_, it can be seen that SiO_2_ has a flat peak at 24°, indicating that SiO_2_ is a non-crystalline solid with an amorphous structure.^23^ For ZIF-8, the peaks of (011), (002), (112), (022), (013) and (222) are the diffraction peaks of ZIF-8.^3^ By comparing the diffraction peaks in the SiO_2_, ZIF-8 and ZIF-8@SiO_2_ XRD patterns, the relative peaks of ZIF-8 and SiO_2_ can be seen clearly in the XRD pattern of ZIF-8@SiO_2_, indicating that ZIF-8@SiO_2_ is synthesized.

#### 3.1.2 FTIR characterization

Fig. S2† shows the FTIR spectra of SiO_2_, ZIF-8 and ZIF-8@SiO_2_. It can be seen from the spectrum of ZIF-8 that the absorption peaks at 3132 cm^−1^ and 2928 cm^−1^ are the asymmetric vibration peaks of the C–H bond in the CH_3_ group and the absorption peak at 1581 cm^−1^ is the stretching vibration of the C–N bond. The absorption peak at 418 cm^−1^ is the stretching vibration mode of the Zn–N bond. The absorption peaks at 500–1500 cm^−1^ belong to the in-plane bending vibration and the stretching vibration of the imidazole ring.^4,24^ From the spectrum of SiO_2_, it can be seen that the absorption peaks at 1096 cm^−1^ and 801 cm^−1^ represent the asymmetric and symmetrical vibration peaks of the Si–O–Si bonds, respectively. And the absorption peak at 472 cm^−1^ belongs to the bending vibration of the Si–O bond.^22,25^ In the spectrum of ZIF-8@SiO_2_, the relative absorption peaks of ZIF-8 and SiO_2_ can be seen clearly, demonstrating that ZIF-8@SiO_2_ is synthesized.

#### 3.1.3 SEM and TEM characterization

**Fig. 1:**
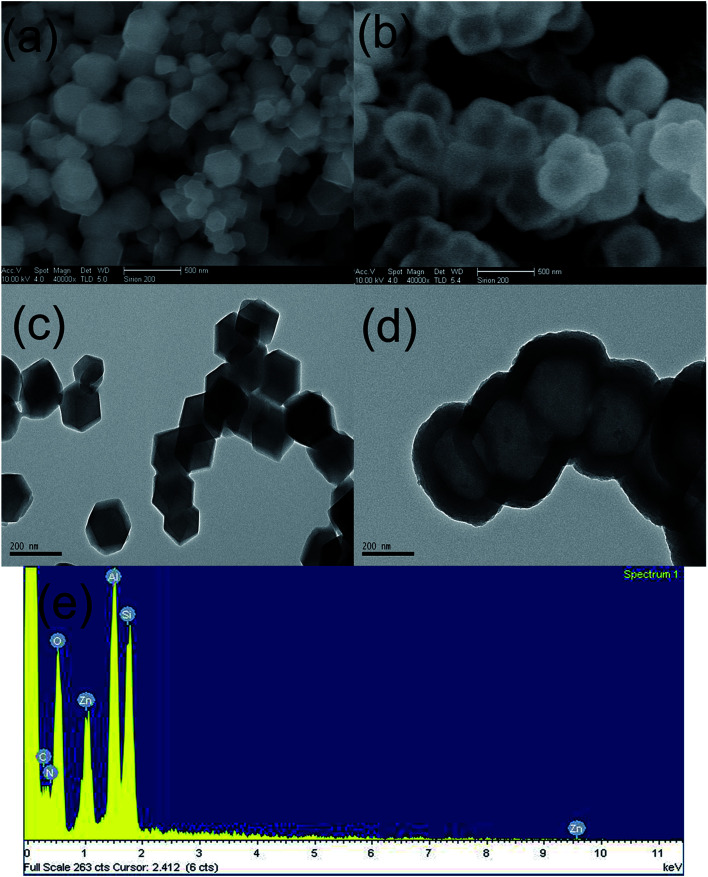
SEM images of (a) ZIF-8 and (b) ZIF-8@SiO_2_, TEM images of (c) ZIF-8 and (d) ZIF-8@SiO_2_, (e) EDS spectrum of ZIF-8@SiO_2_.

Fig. 1(a) and (b) are the SEM images of ZIF-8 and ZIF-8@SiO_2_, respectively. In Fig. 1(a), it can be seen that the synthesized ZIF-8 crystal has a regular rhombic dodecahedral structure, and the distribution of its particle size is uniform. Through comparing Fig. 1(a) and (b), it can be seen that the surface of ZIF-8 is rough and the rhombic dodecahedral structure is not obvious, with an approximate circular structure. This is due to SiO_2_ obtained by the hydrolysis of ethanol solution of TEOS under alkaline conditions, which is involved in the formation of the core–shell structure. Meanwhile, ZIF-8, as the core, forms a layer of SiO_2_ shell on its surface. So it can be ascertained that ZIF-8 is successfully coated with SiO_2_.

In order to further demonstrate that ZIF-8 is coated with SiO_2_ successfully and to more directly observe the structure and morphology of ZIF-8 and ZIF-8@SiO_2_, ZIF-8 and ZIF-8@SiO_2_ are analyzed by TEM. Fig. 1(c) and (d) are the TEM images of ZIF-8 and ZIF-8@SiO_2_, respectively. The rhombic dodecahedral structure of ZIF-8 crystal can be seen more clearly from Fig. 1(c) and the crystal size is the same as in Fig. 1(a) of ZIF-8. It is more intuitive to see the core–shell structure of ZIF-8@SiO_2_ and the surface of ZIF-8 form a layer of SiO_2_ shell. In addition, the crystal structure of ZIF-8 is not destroyed by the overlaying of SiO_2_. The presence of element peaks of C, N, O, Zn and Si can be seen in the EDS of Fig. 1(e) of ZIF-8@SiO_2_, which further demonstrates the successful preparation of the ZIF-8@SiO_2_ core–shell structure nanocrystal. This is consistent with the previous analysis of XRD and FTIR.

#### 3.1.4 XPS characterization of ZIF-8@SiO_2_

XPS is used to analyze the composition of ZIF-8@SiO_2_ and the content of each element. The XPS spectrum of ZIF-8@SiO_2_ is shown in Fig. S3.† The sample contains Si, O, C, N and Zn elements, indicating the synthesis of ZIF-8@SiO_2_. The surface composition of ZIF-8@SiO_2_ is of Si and O elements, and its related characteristic peaks are clearly visible on the XPS survey spectrum. In addition, the percentage of Si and O elements is 15.6% and 41.4%. Combined with the results of XRD, FTIR, SEM and TEM, it indicates that the core–shell structure of ZIF-8@SiO_2_ is successfully synthesized.

#### 3.1.5 TG characterization

The thermal degradation behavior of SiO_2_, ZIF-8 and ZIF-8@SiO_2_ is studied by thermogravimetric analysis under air condition. The TGA curves of SiO_2_, ZIF-8 and ZIF-8@SiO_2_ are shown in Fig. S4.† For SiO_2_, the thermal degradation process has three stages. The first stage is due to the physical adsorption of water and solvent (room temperature-110 °C), the second stage is due to the polycondensation of the structure of the silica network (110–220 °C), and the third stage is attributed to the condensation and dehydration of silanol groups (220–360 °C).^16,26,27^ The weight loss of ZIF-8 also has three stages. The first stage is the evaporation of the methanol molecules adsorbed on the surface of ZIF-8 below 100 °C. Secondly, the degradation is due to the carbonization of 2-methylimidazole molecules in the pores of ZIF-8 between 100 °C and 350 °C. The final stage of weight loss beginning from 350 °C is caused by the decomposition of the organic groups and ZIF-8 nanocrystals.^28,29^ From the TGA curve of ZIF-8@SiO_2_, it can be seen that the weight loss is only 22.4%, which shows that ZIF-8 after SiO_2_ coating can obviously enhance the thermal stability of ZIF-8.

### 3.2 Characterization of EP and EP composites

#### 3.2.1 Thermal stability of EP composites

**Fig. 2:**
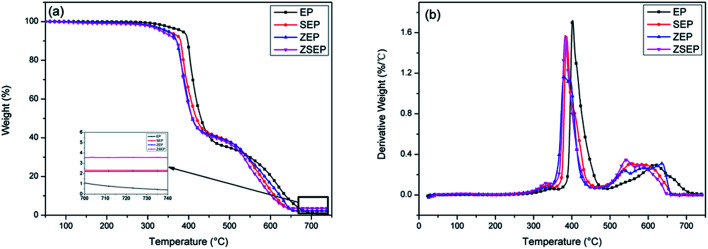
TGA (a) and DTG (b) curves of neat EP and EP composites.

The thermostability and thermal degradation behavior of EP and EP composites are investigated by thermogravimetric analysis under air condition. The TGA and derivative thermogravimetric (DTG) curves are shown in Fig. 2, and the relevant data is shown in Table 2. *T*_10%_, *T*_50%_ and *T*_max_ in Table 2 represent the temperature at 10 wt% mass loss, 50 wt% mass loss and the maximum thermal decomposition rate, respectively. The *T*_10%_, *T*_50%_ and *T*_max_ of SEP, ZEP and ZSEP composites are decreased to some extent compared with those of EP, indicating that the degradation of EP has a different degree of catalytic effect. This is because the surface of SiO_2_ has many silanol groups which can be used as Brønsted acid sites with a role of catalytic degradation of polymers.^16,30^ In addition, ZIF-8 contains Zn, and Zn as one of the transition metal elements has an effect on catalyzing the degradation of polymer.^31^ However, it is worth noting that, compared with those of ZEP, the *T*_10%_, *T*_50%_ and *T*_max_ of ZSEP are increased to some extent, which indicates that, after SiO_2_ coating, ZIF-8 could improve the high temperature thermal stability of the composites.

**Table 2:** TG data of neat EP and EP composites

Sample	*T*_10%_ (°C)	*T*_50%_ (°C)	*T*_max_ (°C)	Char yield (%)
EP	397.5	430.2	402.2	0.4
SEP	380.2	418.9	385.6	2.3
ZEP	370.4	408.3	380.5	2.2
ZSEP	371.5	409.4	383.2	3.6

It can be seen from the char residue of the TG test that the char residue of pure EP is only 0.4% at 750 °C. Compared with that of EP, the char residue of all the composites are improved to some extent, as shown in Table 2. Among them, ZSEP has the highest rate of char residue, reaching 3.6%. This is because SiO_2_ can not only play the role as a physical barrier, but can also play a role in strengthening the catalytic activity of metal oxides, which further promotes the formation of a char layer.^14^

**Fig. 3:**
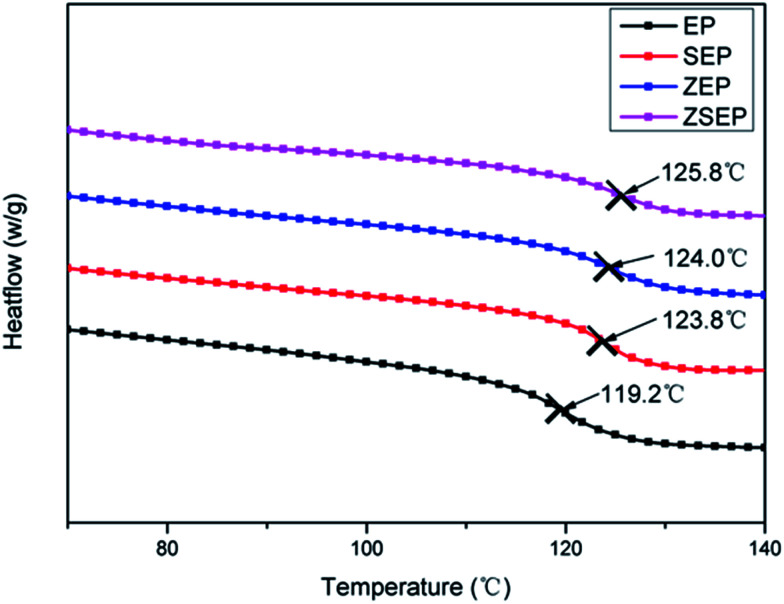
DSC curves of neat EP and EP composites.

It is well known that the glass transition temperature (*T*_g_) is an important thermal parameter for studying the motion of polymer segments. The effects of SiO_2_, ZIF-8 and ZIF-8@SiO_2_ on the *T*_g_ of EP are investigated by DSC. Fig. 3 shows the DSC curves for neat EP and EP composites. When SiO_2_, ZIF-8 and ZIF-8@SiO_2_ are added to EP, the *T*_g_ of the composites is increased by 4–6 °C. However, the change in the *T*_g_ is not significant by comparisons between the composites. When 2 wt% ZIF-8@SiO_2_ is added to EP, the *T*_g_ of EP is increased from 119.2 °C to 125.8 °C. This is due to the interfacial interaction between ZIF-8@SiO_2_ and EP, so ZIF-8@SiO_2_ as a physical barrier, hinders the chain motion of the polymer.

#### 3.2.2 Flame retardancy of neat EP and EP composites

**Fig. 4:**
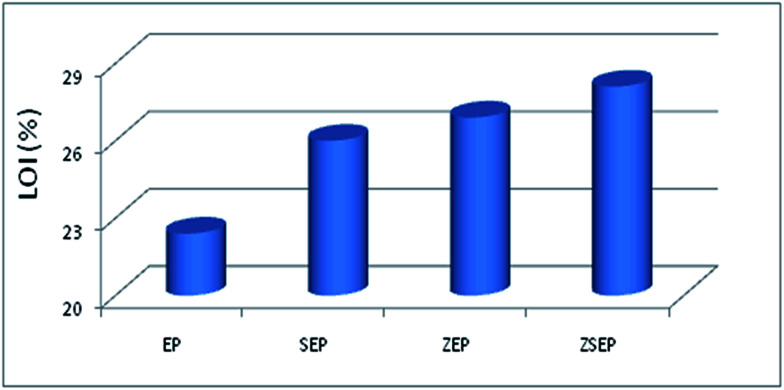
LOI values of neat EP and EP composites.

The limiting oxygen index refers to the volume fraction of oxygen in the oxygen and nitrogen mixed gas when it is capable of supporting its combustion. The LOI values of neat EP and EP composites are shown in Fig. 4 and Table 3. The value of LOI of neat EP is 22.4%, indicating that pure EP is easy to burn in air. Compared with that of EP, the values of LOI of SEP, ZEP and ZSEP increase in varying degrees. It can be seen from the data of LOI that the LOI of ZSEP increases to 28.1%, indicating that ZSEP has the best flame retardancy in comparison with EP, SEP and ZEP.

**Table 3:** The data from cone calorimeter and LOI test of neat EP and EP composites

Sample	PHRR (kW m^−2^)	THR (MJ m^−2^)	SPR (m^2^ s^−1^)	TSP (m^2^)	LOI (%)
EP	1054	39.1	0.88	67.8	22.4
SEP	727	34.4	0.63	61.9	26.0
ZEP	431	25.3	0.69	52.2	26.9
ZSEP	254	23.9	0.43	40.8	28.1

**Fig. 5:**
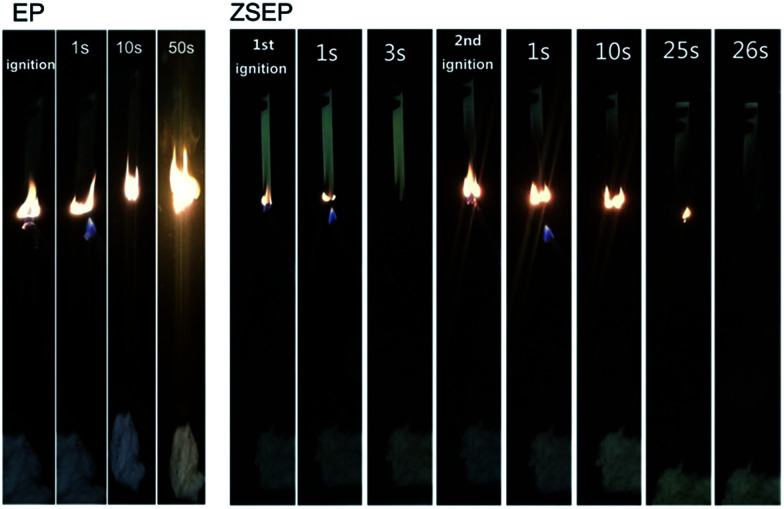
Combustion processes of neat EP and ZSEP during the UL-94 vertical burning test at different times.

At the same time, EP and EP composites are measured by UL-94 vertical combustion. The results show that SEP and ZEP have no level. However, ZSEP with 2 wt% ZIF-8@SiO_2_ added can reach the V-1 level. Fig. 5 shows the images of EP and ZSEP during the combustion process. All those indicate that ZIF-8@SiO_2_ has better flame retardancy.

**Fig. 6:**
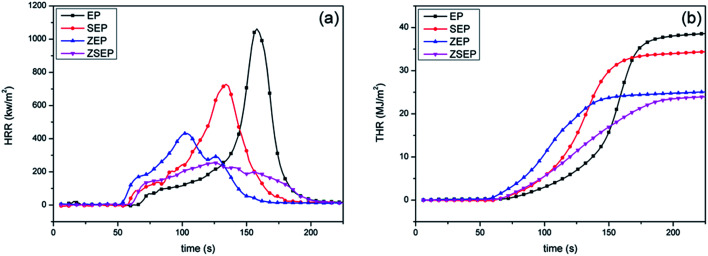
HRR (a) and THR (b) curves of neat EP and EP composites.

The cone calorimeter may make one of the most ideal experimental tests used to characterize the combustion performance of materials. In order to study the actual combustion of EP and EP composites, the samples are measured by cone calorimeter. The combustion curves and specific data are shown in Fig. 6 and Table 3. In Fig. 6(a), the peak heat release rate (PHRR) can reach 1054 kW m^−2^ when pure EP is burned, indicating that EP is easy to burn when there is a heat source. Compared to that of EP, the PHRR of SEP is decreased by 31.0%. This is mainly because SiO_2_ is easy to migrate to the surface of a polymer during the combustion process, and acts as a physical barrier to isolate oxygen and suppress the volatilization of the combustible gases produced by the polymer during combustion, thereby reducing the heat release rate. Compared to that of EP, the PHRR of ZEP is decreased by 59.1%. This is mainly because ZIF-8 is a N-containing material, and N-containing materials in the combustion process may release NH_3_ and N_2_ which will dilute the oxygen and the concentration of combustible gas, so as to achieve the purpose of flame retardancy.^9^ At the same time, ZIF-8 will form a metal oxide during the combustion process and can catalyze the polymer cross-linked into carbon, so that the stability of the char layer has a better barrier effect,^32^ and achieve better flame retardancy. It is worth noting that compared with that of EP, the PHRR of ZSEP decreases by 75.9%.

From Fig. 6(b), the total heat release (THR) of neat EP is 39.1 MJ m^−2^. The THR of all EP composites is decreased in different degrees in comparison with that of neat EP, and the THR of SEP, ZEP and ZSEP decrease by 12.0%, 35.3% and 38.9%, respectively. Among them, the THR of ZSEP is decreased most obviously. This is because, in the process of combustion, the generation of SiO_2_ can enhance the catalytic effect of metal oxide decomposed by ZIF-8, which can effectively promote the formation of decomposition products of the polymer into char, forming a better char layer to isolate heat and oxygen delivery, prevent the further combustion of the polymer matrix, and effectively improve the flame retardancy of the composites.

#### 3.2.3 Smoke suppression of neat EP and EP composites

**Fig. 7:**
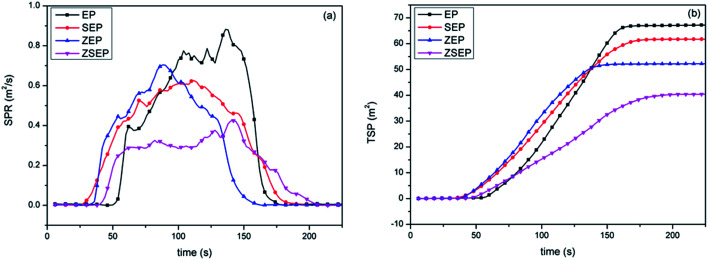
SPR (a) and TSP (b) curves of neat EP and EP composites.

The smoke production rate (SPR) and total smoke production (TSP) can be used as an evaluation index of smoke release during combustion. The SPR, TSP curves and specific data are shown in Fig. 7(a) and (b) and Table 3. It can be seen from the SPR curves in Fig. 7(a) that the SPR of neat EP is 0.88 m^−2^ s^−1^ during combustion, indicating that neat EP will release a large amount of smoke dust in the combustion process. By comparing the EP composites with pure EP, it is found that the SPR of all the EP composites decreases in varying degrees. Compared with that of neat EP, the SPR of SEP and ZEP are decreased by 28.4% and 21.6%, respectively. For SEP, the addition of SiO_2_ can form a covering layer on the surface of the polymer to play a physical barrier role. For ZEP, smoke suppression is due to the ZIF-8 thermal decomposition of metal oxides during the combustion process, metal oxides have a large specific surface area that can not only absorb smoke dust, but also catalyze the polymer cross-linked into char to form a char layer to achieve the effect of smoke suppression.^33^ However, it is worth noting that the SPR of ZSEP decreases more significantly in comparison with those of ZSEP and SEP.

From the TSP curves in Fig. 7(b), it can be seen that the TSP of pure EP can reach 67.8 m^2^. Compared with that of neat EP, the TSP of SEP, ZEP and ZSEP are decreased by 8.7%, 23.0% and 39.8%, respectively. Obviously, the TSP of ZSEP falls the most. This is because ZSEP could generate SiO_2_ and metal oxides during the combustion process, SiO_2_ covers the surface of the polymer matrix and plays a role of a physical barrier. At the same time, metal oxide's catalytic activity will be further enhanced in the presence of SiO_2_, and promote the formation of char residue which may form a denser char layer that inhibits the decomposition of the polymer and reduces the amount of smoke released, resulting in a better smoke suppression effect.

### 3.3 Char residue analysis

#### 3.3.1 LRS analysis of char residue

**Fig. 8:**
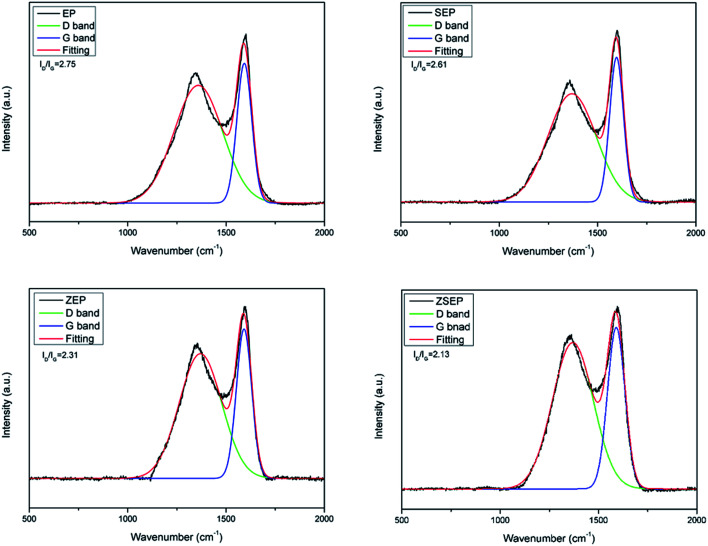
Raman spectra of char residue of EP and EP composites.

Laser Raman spectroscopy is an effective method to study the properties of char residue formed by the combustion of a polymer. In order to further analyze the effect of SiO_2_, ZIF-8 and ZIF-8@SiO_2_ on the flame retardancy of EP, the char residue of the EP composites is analyzed by LRS. Fig. 8 shows the LRS spectra of the char residue of neat EP and EP composites, and the peaks are fitted by the Gaussian method. All samples have two distinct absorption bands, a peak near 1580 cm^−1^ (G peak), and another near 1350–1380 cm^−1^ (D peak). G peak is the 2E_2g_ vibration of C–C bond in graphite, which represents sp^2^ hybridization; D peak is the mode of vibration of carbon atom, which represents sp^3^ hybridization. The *I*_D_/*I*_G_ can be used to measure the degree of graphitization of the char layer, where *I*_D_ and *I*_G_ represent the relative areas of the D and G peaks, respectively. The smaller the value of *I*_D_/*I*_G_, the higher the degree of graphitization of the char layer. In other words, when the char residue has a smaller sp^3^/sp^2^ hybridization ratio, it can form smaller microcrystalline graphite, thus facilitating the formation of a more compact char layer. This compact char layer provides good insulation and oxygen barrier to effectively block the degradation of materials and the spread of the flame to achieve the purpose of improving the flame retardancy properties of composite materials.^34,35^ As can be seen in Fig. 8, the *I*_D_/*I*_G_ values of EP, SEP, ZEP and ZSEP are 2.75, 2.51, 2.31 and 2.13, respectively, decreasing by 8.7%, 16.0% and 22.5%, respectively, compared with that of EP. It shows that ZSEP has the highest degree of graphitization of char residue and the best thermal stability.

#### 3.3.2 XPS analysis of char residue

**Fig. 9:**
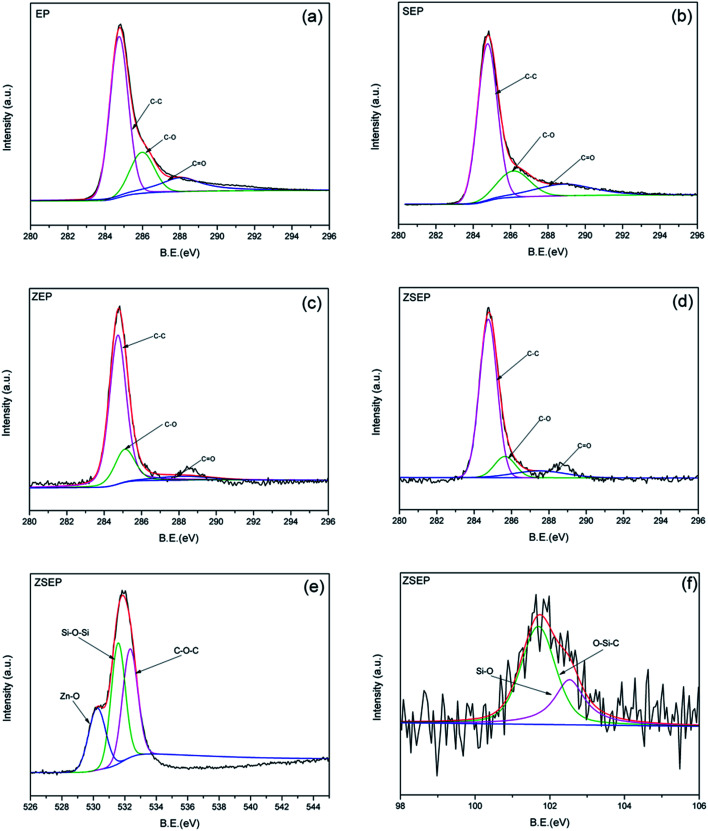
C1s spectra of char residue of EP and EP composites: EP (a), SEP (b), ZEP (c) and ZSEP (d), O1s spectrum of ZSEP (e), Si_2p_ spectrum of ZSEP (f).

XPS is used to analyze the char residue of EP and EP composites after the combustion of cone calorimeter. Fig. 9(a)–(d) are the C1s spectra of EP, SEP, ZEP and ZSEP char residue, respectively. C–C, C–O and C=O represent the C atom of the aliphatic and aromatic structures, the C atom of the hydroxyl group or the ether bond and the C atom of the carbonyl group, respectively. The peaks at 284.7 eV, 285.9 eV and 288.2 are ascribed to C–C, C–O and C=O, respectively. The calculated values for Cox/Ca in Table 4 are used to study the thermal oxidation resistance of char. And the content of the oxidized carbon atoms (C–O, C=O) is represented by Cox, and Ca is used to represent the content of non-oxidized carbon atoms (C–C, C–H). The smaller the value of Cox/Ca, the higher the thermal oxidation resistance of the material which is easy to form a char layer during combustion.^36^ In Table 4, it can be seen that the values of Cox/Ca for EP, SEP, ZEP and ZSEP are 0.69, 0.54, 0.34 and 0.29, respectively. Compared with that of neat EP, the values of Cox/Ca of all the EP composites are decreased in different degrees, but it is worth noting that of ZSEP is reduced by 58.0%. This is because the formation of a dense char layer on the surface of polymer prevents the contact of the internal material with outside oxygen during the combustion of ZSEP, which effectively reduces the degree of oxidation of the internal material. In addition, Si and Zn are combined with more oxygen atoms to facilitate the formation of a stable char layer. The O1s and Si_2p_ spectra of ZSEP char residue are shown in Fig. 9(e) and (f), respectively. In the O1s spectrum, the peaks at 530.2 eV, 531.7 eV and 533 eV represent the peaks of Zn–O, Si–O–Si and C–O–C, respectively.^9,37^ And for the Si_2p_ spectrum, the peaks at 101.8 eV and 102.5 eV belong to O–Si–C and Si–O bonds.^38,39^ This indicates that Si elements migrate to the surface of the polymer matrix to form Si–O–Si structure of the char layer to protect the internal polymer of ZSEP in the combustion process. In addition, the formation of compounds which contain O–Si–C bond is also beneficial to increasing the thermal stability and strength of the char layer. The appearance of Zn–O bond indicates that the char residue may contain zinc oxide. In a previous study,^14^ SiO_2_ not only catalyzed the degradation of polymers, but also more efficiently catalyzed the degradation of the polymer into char when metal oxides were present, contributing to the formation of a dense char layer. Therefore, the synergistic effect of SiO_2_ and ZnO is helpful in improving the flame retardancy and smoke suppression properties of the polymer.

**Table 4:** Results of C1s XPS of char residue of neat EP and EP composites

Sample	C–C area (%)	C–O area (%)	C=O area (%)	Cox/Ca
EP	59.3	21.7	19	0.69
SEP	65.1	17.8	17.1	0.54
ZEP	74.9	19.8	5.3	0.34
ZSEP	77.6	12.4	10.0	0.29

#### 3.3.3 SEM and EDS analysis of char residue

**Fig. 10:**
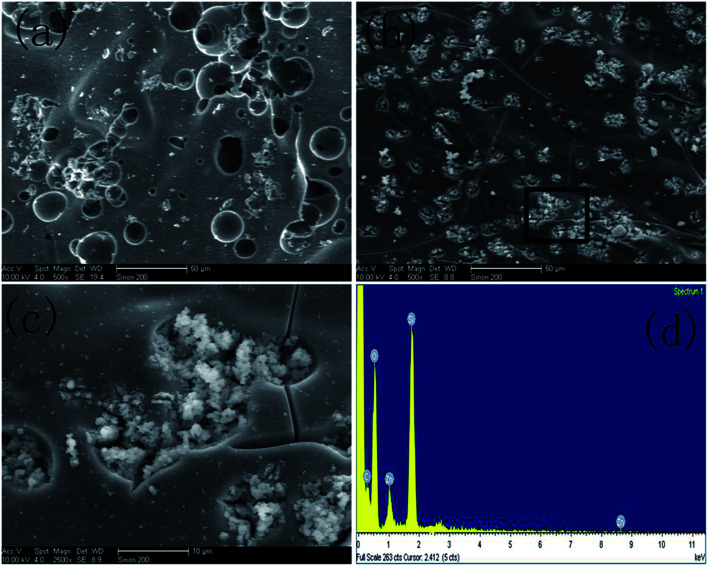
SEM images of char residue of (a) EP and (b) ZSEP, (c) is a partially enlarged image of the black rectangle in (b), (d) EDS spectrum of char residue of ZSEP.

In order to further explore the mechanism of flame retardancy and smoke suppression, the char residue of EP and ZSEP is investigated by SEM detection after cone calorimeter combustion. The micromorphology of the char residue of EP and ZSEP is shown in Fig. 10(a) and (b), respectively. The surface of char residue of pure EP has a large number of holes. It can be seen from Fig. 10(b) that the char residue of ZSEP is more continuous and dense than EP, and can effectively inhibit the exchange of heat and combustible gas between the condensed phase and gas phase. Fig. 10(c) is obtained by partial enlargement in the black box of Fig. 10(b). Fig. 10(c) exhibits that the surface of ZSEP char residue has a lot of granular materials covered in the above, which can play a good physical barrier effect. In addition, EDS detection of ZSEP char residue shows that the char residue contains C, O, Si and Zn elements, indicating that the char residue may contain SiO_2_ and ZnO. Due to the synergistic effect of SiO_2_ and ZnO, the char layer is more continuous and dense and achieves a better flame retardancy and smoke suppression effect.

#### 3.3.4 FTIR and XRD analysis of char residue

**Fig. 11:**
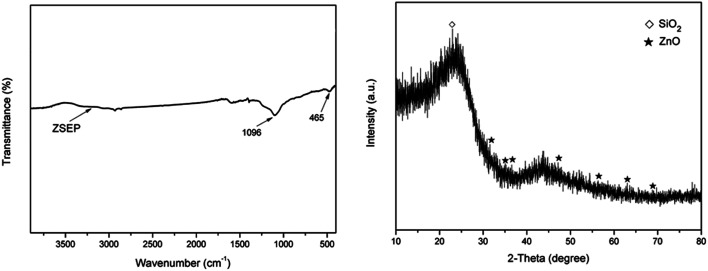
FTIR and XRD spectra of char residue of ZSEP.

The FTIR spectrum and XRD pattern of char residue of ZSEP after the cone calorimeter combustion are shown in Fig. 11. It can be seen from the FTIR spectrum that the absorption peak at 1096 cm^−1^ is the stretching vibration peak of Si–O–Si, indicating that SiO_2_ still exists in the char residue of the ZSEP composite after combustion by cone calorimeter. The absorption peak at 465 cm^−1^ is caused by the stretching vibration of the Zn–O bond after the combustion of zinc–silicon nanomaterials,^40^ which indicates that the char residue of the ZSEP composite is likely to contain ZnO. At the same time, the characteristic peaks of SiO_2_ and ZnO can also be seen in the XRD pattern of the char residue,^41^ which further proves that ZSEP could produce SiO_2_ and ZnO during the combustion process. SiO_2_ not only has the effect of a physical barrier, but also synergistically catalyzes into char with metal oxide ZnO to improve the density of the char layer, thereby enhancing the flame retardancy and smoke suppression of the composite material.

### 3.4 Mechanism of flame retardancy and smoke suppression

Based on the above research, the mechanism of ZIF-8@SiO_2_ to improve the flame retardancy and smoke suppression of the EP composite materials is briefly described as follows. There are three main reasons for the improvement of flame retardancy and smoke suppression performance of the EP composites. Firstly, ZSEP could produce SiO_2_ during the combustion process, which migrates to the surface of the polymer matrix to form a covering layer, accompanied by the formation of compounds that contain Si–O–Si and O–Si–C structures, which act as a physical barrier to the polymer matrix, protect the internal material and improve its thermal stability. Secondly, ZIF-8 as an N-containing material may release NH_3_ and N_2_ during the combustion process, and NH_3_ and N_2_ dilute oxygen and the concentration of combustible gas decomposed by EP, so as to achieve a flame retardant effect. In addition, the decomposition of ZIF-8 produces ZnO, which promotes the formation of a char layer. Finally, SiO_2_ is a solid acid with more acidic active sites which promote carbonization by synergistic catalysis with metal oxide, and thus improve the density of the char layer and further improve the flame retardancy and smoke suppression performance of the composite.

## 4. Conclusions

In this study, a novel material with a core–shell structure of ZIF-8@SiO_2_ was synthesized by the sol–gel method. The structure, morphology and composition of ZIF-8@SiO_2_ were characterized by XRD, FT-IR, SEM-EDS, TEM and XPS. Results showed that the ZIF-8@SiO_2_ core–shell structured material was successfully synthesized. 2 wt% ZIF-8@SiO_2_ was added into EP to study its thermal stability, flame retardancy and smoke suppression. The results showed that the addition of ZIF-8@SiO_2_ to EP could significantly improve the char yield. Moreover, the value of LOI reached 28.1% and the UL-94 vertical combustion reached the V-1 level. At the same time, the PHRR, THR, SPR and TSP decreased by 75.9%, 38.9%, 51.1% and 39.8%, respectively. The analysis of char residue led to a better understanding of the mechanism of flame retardancy and smoke suppression as a result of the physical barrier of SiO_2_, the gas phase flame retardancy of inert gas decomposed by ZIF-8 and the synergistic effect of SiO_2_ and ZnO that jointly enhanced the flame retardancy and smoke suppression performance of the EP composite.

## Conflicts of interest

There are no conflicts to declare.

## Supplementary Material

RA-008-C7RA12816A-s001
