# Supplementary material for: Zeolitic imidazolate framework-8 was coated with silica and investigated as a flame retardant to improve the flame retardancy and smoke suppression of epoxy resin
Source: RSC Adv. 2018 Jan 11;8(5):2575–85. doi: 10.1039/c7ra12816a (PMC9077404; doi:10.1039/c7ra12816a)
Supplement: RA-008-C7RA12816A-s001 [file RA-008-C7RA12816A-s001.pdf]

Electronic Supplementary Material (ESI) for RSC Advances.
This journal is © The Royal Society of Chemistry 2018

Supporting information

Zeolitic imidazolate framework-8 was coated with silica and as a flame retardant to improve the flame retardancy and smoke suppression of epoxy resin

Wenzong Xu, [*,†] **Guisong Wang,** [†] **Yucheng Liu,** [†] **Rui Chen** [†] **and Wu Li**[†]

[†]School of Materials Science and Chemical Engineering, Anhui Jianzhu University, 292 Ziyun Road, Hefei, Anhui 230601, People's Republic of China

*Correspondence to: Wenzong Xu

(Tel./Fax:+86-0551-63828157.   Email: wenzongxu@ahjzu.edu.cn)

The content of the supporting information:

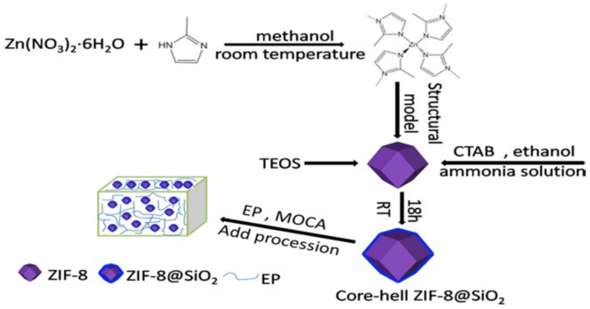

Scheme S1. Illustration of synthesis of ZIF-8@SiO_2 and preparation of EP composites.

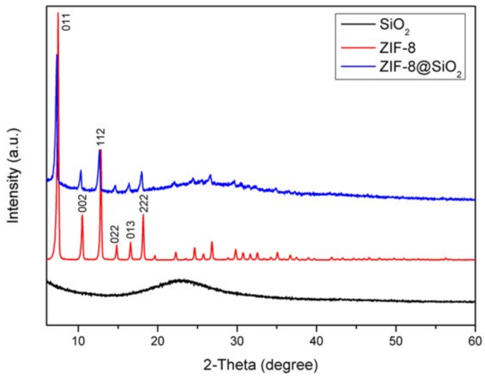

Figure S1. XRD patterns of SiO_2, ZIF-8 and ZIF-8@SiO_2.

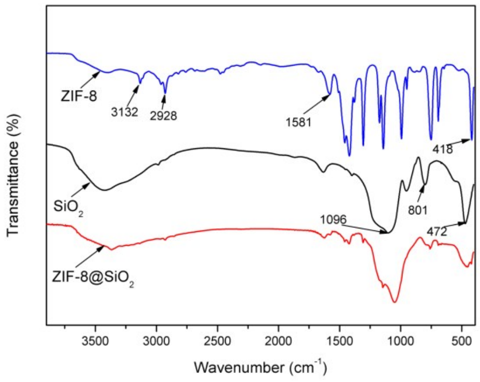

Figure S2. FTIR spectra of SiO_2, ZIF-8 and ZIF-8@SiO_2.

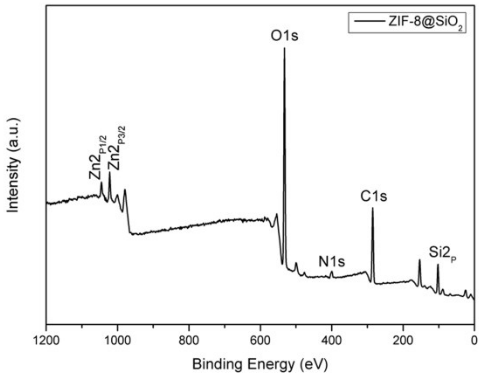

Figure S3. XPS spectrum of ZIF-8@SiO_2.

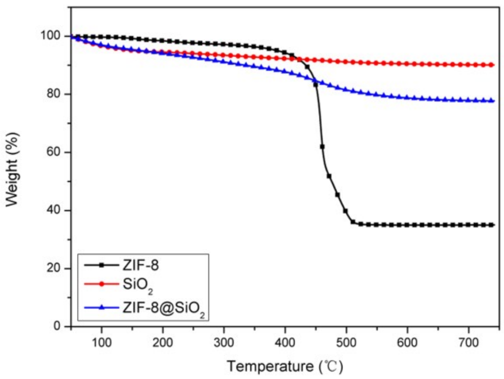

Figure S4. TG curves of SiO_2, ZIF-8 and ZIF-8@SiO_2.
